# HIF-1α Induces HECTD2 Up-Regulation and Aggravates the Malignant Progression of Renal Cell Cancer *via* Repressing miR-320a

**DOI:** 10.3389/fcell.2021.775642

**Published:** 2021-12-24

**Authors:** Dong Lv, Taimin Shen, Juncheng Yao, Qi Yang, Ying Xiang, Zhiwei Ma

**Affiliations:** ^1^ Department of Urology, Eastern Hospital, Sichuan Academy of Medical Sciences and Sichuan Provincial People’s Hospital, Chengdu, China; ^2^ Health Management Center, Sichuan Academy of Medical Sciences and Sichuan Provincial People’s Hospital, Chengdu, China; ^3^ Department of Urology, Sichuan Academy of Medical Sciences and Sichuan Provincial People’s Hospital, Chengdu, China

**Keywords:** HIF-1α, HECTD2, MiR-320a, renal cell carcinoma, progression

## Abstract

Renal cell carcinoma (RCC) is a frequent malignancy of the urinary system. It has been found that hypoxia mediates the malignant evolvement of RCC. Here, we probe the impact and potential mechanism of HECT domain E3 ubiquitin-protein ligase 2 (HECTD2) and HIF-1α on regulating RCC evolvement. RCC tissues and adjacent normal tissues were collected, and the association between the expression profiles of HECTD2 and HIF-1α and the clinicopathological features was analyzed. Additionally, we constructed HECTD2/HIF-1α overexpression and knockdown models in RCC cell lines to ascertain the impacts of HECTD2 and HIF-1α on RCC cell proliferation, apoptosis, migration, and growth *in vivo*. We applied bioinformatics to predict the upstream miRNA targets of HECTD2. Meanwhile, RNA immunoprecipitation (RIP), and the dual-luciferase reporter assays were employed to clarify the targeting association between HECTD2 and miR-320a. The effect of miR-320a on HECTD2-mediated RCC progression was investigated. The results suggested that both HIF-1α and HECTD2 were up-regulated in RCC (compared with adjacent non-tumor tissues), and they had positive relationship. Moreover, higher level of HECTD2 and HIF-1α is associated with poorer overall survival of RCC patients. HECTD2 overexpression heightened RCC cell proliferation and migration, and weakened cell apoptosis. On the other hand, the malignant phenotypes of RCC cells were signally impeded by HECTD2 or HIF-1α knockdown. Moreover, miR-320a targeted the 3′-untranslated region of HECTD2 and suppressed HECTD2 expression. The rescue experiments showed that miR-320a restrained HECTD2-mediated malignant progression in RCC, while up-regulation of HIF-1α hampered miR-320a expression. Collectively, HIF-1α mediated HECTD2 up-regulation and aggravated RCC progression by attenuating miR-320a.

**GRAPHICAL ABSTRACT:**
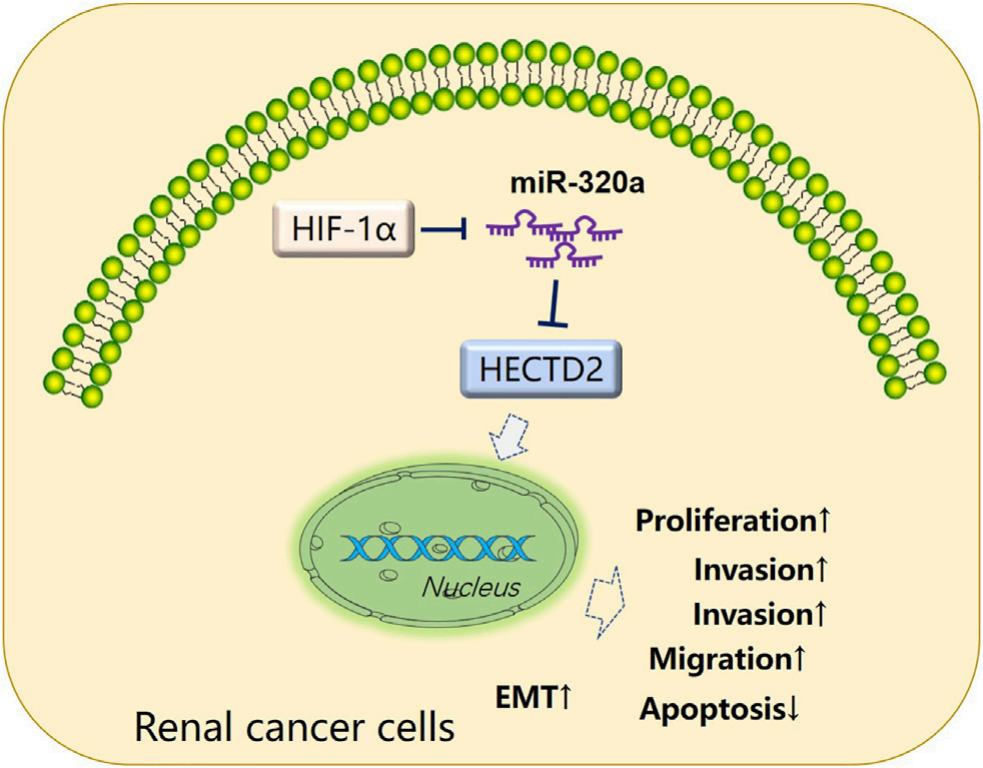


Graphical abstract: HIF-1α enhances HECTD2 expression by attenuating miR-320a, thereby boosting RCC cell proliferation, migration, invasion, and EMT and abating apoptosis, which accelerates the malignant progression of RCC.

## Introduction

Renal cell cancer (RCC) is the third most familiar genitourinary malignancy globally, with a high mortality rate (Shingarev and Jaimes, 2017). Clear cell renal cell carcinoma (ccRCC) is a familiar subtype of RCC, accounting for approximately 75% of all RCC cases (Song et al., 2018; Wolf et al., 2020). Over the last few years, treatment of RCC has improved with advances in medical technology, but the prognosis of RCC patients remains poor due to its high recurrence and metastasis rate (Wu et al., 2014; Di Franco et al., 2020). Therefore, it’s indispensable to further probe the molecular regulatory mechanism of the occurrence and development of RCC and provide new biomarkers for the targeted therapy.

microRNAs (miRNAs) are processed from pri-miRNAs. They are highly conserved endogenous single-stranded non-coding small RNAs with small fragments of 20–24 nucleotides in length (Saliminejad et al., 2019). Studies have manifested that miRNA functions as a post-transcriptional regulator that can modulate cancer evolvement by combining with specific sequences of 3′UTR of the target genes and impeding the expression of these genes (Ferragut Cardoso et al., 2020). It is reported that miRNAs are extensively involved in RCC development. For example, it has been revealed that miR-340 is up-regulated in RCC, which accelerates RCC cell proliferation, migration and invasion by hampering the FZD3 profile (Xiang et al., 2021). Additionally, miR-31-5p is lowly expressed in RCC, and it attenuates the malignant behaviors of RCC cells by targeting and reversely modulating HMGA1 (Liu et al., 2021). Also, Cheng C et al. have found that miR-26a-5p is knocked down in RCC, and it curbs RCC cell proliferation and invasion and intensifies cell death by targeting E2F7 (Cheng et al., 2020). miR-320a has been found sponged by lncRNA CCAT2 (Wu et al., 2020), but its downstream mechanism in RCC progression needs further investigation.

Hypoxia is among the usual pathological conditions in which oxygen is restricted in tissues, and it facilitates tumor cell invasion and migration, impedes apoptosis, and is associated with tumor metastasis (Rankin et al., 2016). RCC, originating from renal proximal tubular epithelial cells, is one of the most frequent cancers that are highly sensitive to hypoxia (Schödel et al., 2016). Hypoxia-inducible factor 1α (HIF-1α), a functional subunit of HIF-1, has been confirmed to be extensively involved in RCC evolvement as an essential transcription factor that exerts an active role in hypoxia (Gudas et al., 2014). Besides, it has been revealed that apoptosis inhibition induces cancer cell proliferation and metastasis under hypoxia (Ge et al., 2018). Homologous to E6AP C-terminus (HECT) E3 ubiquitin ligase (HECTD) members have been found involved in tumor progression through ubiquitin modification (Jiang et al., 2020). For example, HECTD1 downregulation enhances cervical cell migration and epithelial-mesenchymal transition by stabilizing SNAIL in a ubiquitination regulation manner (Wang et al., 2020). HETCD2 is a kind of E3 ubiquitin ligase located at 10q23.32, with 4,874 bp in length. Similar to the other E3 ubiquitin ligases, HECTD2 ubiquitinated and induced protein inhibitor of activated STAT 1 (PIAS1), thus contributing to innate immunity and experimental lung injury followed by pneumonia (Coon et al., 2015). More importantly, HECTD2 also participates in neuroblastoma evolvement (Suo et al., 2018). Nevertheless, further research is needed to determine whether HECTD2 has any significant value in RCC.

By detecting the expression of HIF-1α and HECTD2 in RCC tissues and cells, we discovered that HIF-1α and HECTD2 were overexpressed in RCC and had a positive relationship. HIF-1α boosted HECTD2 expression and facilitated the malignant biological behaviors of RCC. In addition, we adopted bioinformatics analysis and the result revealed a targeted relationship between miR-320a and HECTD2, whereas HIF-1α inhibited miR-320a. Thus, we supposed that there’s a novel HIF-1α/miR-320a/HECTD2 axis in RCC progression. In conclusion, this study uncovers a new molecular mechanism in RCC and provides a new theoretical reference for RCC therapy.

## Materials and Methods

### Patient Tissue Samples

This experiment was authorized by the Ethics Committee of Sichuan Provincial People’s Hospital and implemented in accordance with the Helsinki Declaration. RCC tissues were obtained from 104 RCC patients (45.0 ± 12.3 years old) who underwent surgical excision but did not receive chemotherapy or radiotherapy. The matched paracancerous normal tissues were also obtained from the same patients, which were 3–4 cm away from the tumorous tissues, and no cancer cells were discovered in the postoperative pathological examination. All tissues were frozen in liquid nitrogen and stored at 80°C for subsequent testing. All patients participating in this study signed informed consent. The general information of patients is shown in Tables 1, 2.

**TABLE 1:** Association between the expression of HECTD2 and clinical features in tissue samples of patients with RCC.

Characteristics	Patients	Expression of HECTD2		*p*-value
		Low-HECTD2	High-HECTD2	
Total	104	49	55	—
Age (years)	—	—	—	0.599
<60	58	26	32	—
≥60	46	23	23	—
Gender	—	—	—	0.207
Male	66	28	38	—
Female	38	21	17	—
Tumor stage	—	—	—	0.021*
pT1	47	28	19	—
pT2/T3	57	21	36	—
Fuhrman grade	—	—	—	0.034*
I + II	48	28	20	—
III + IV	56	21	35	—
Tumor diameter (cm)	—	—	—	0.451
≤7 cm	55	24	31	—
>7 cm	49	25	24	—
Lymph node metastasis	—	—	—	0.209
Negative	68	29	39	—
Positive	36	20	16	—

**TABLE 2:** Correlation between HIF-1α expression and clinical features in RCC patients.

Characteristics	Patients	Expression of HIF-1α		*p*-value
		Low- HIF-1α	High-HIF-1α	
Total	104	53	51	—
Age (years)	—	—	—	0.876
<60	64	33	31	—
≥60	40	20	20	—
Gender	—	—	—	0.698
Male	53	28	25	—
Female	51	25	26	—
Tumor stage	—	—	—	0.048*
pT1	23	30	19	—
pT2/T3	19	23	32	—
Fuhrman grade	—	—	—	0.049*
I + II	51	31	20	—
III + IV	53	22	31	—
Tumor diameter (cm)	—	—	—	0.708
≤7 cm	57	30	27	—
>7 cm	47	23	24	—
Lymph node metastasis	—	—	—	0.838
Negative	50	26	24	—
Positive	54	27	27	—

(Note: *p < 0.05, the difference was statistically significant).

### Cell Culture and Transfection

RCC cell lines 786-O and A-498 were bought from the America Type Culture Collection (ATCC, Manassas, VA, United States). Cells were grown in the DMEM (Thermo Fisher Scientific, Waltham, MA, United States) comprising 10% fetal bovine serum, 100 U/mL penicillin (Invitrogen, Carlsbad, CA, United States) and 100 μg/ml streptomycin (Invitrogen) and maintained at 37°C in a 5% CO_2_ incubator. Cells in the logarithmic growth stage were trypsinized before being seeded into 6-well plates (5 × 10^6^ cells/well). The transfection was made after cell growth was stable. 786-O and A-498 cells were transfected with HECTD2 overexpression and knockdown plasmids, HIF-1α overexpression plasmids, miR-320a mimics/inhibitors and the corresponding negative controls. Afterward, they were incubated at 37°C with 5% CO_2_. After transfection for 24 h, the cells were employed for subsequent experiments. The knockdown sequences of HECTD2: si-HECTD2, AUA​AAA​CUG​CUG​AAA​GUG​GAA; si-NC: TTC​TCC​GAA​CGT​GTC​ACG​T.

### Colony Formation Assay

Cells were inoculated into 6-well plates (500 cells/well), and the culture solution was altered every 5 days. After incubation for 7–14 days, cells were rinsed with PBS, immobilized with 4% paraformaldehyde, and dyed with 0.5% crystal violet. At last, the colony formation number was counted using Olympus microscope (BX23, Japan).

### 5-Bromodeoxyuridine (BrdU) Staining

The BrdU cell proliferation detection kit (Wuhan AmyJet Scientific Inc. Wuhan, China) was applied for cell proliferation assessment. 786-O and A-498 cells were seeded into 96-well plates (6 × 10^4^ cells/mL), respectively and incubated overnight with the constant temperature at 37°C and 5% CO_2_ environment. The next day, each well was supplemented with 20 μL BrdU (50 mM) and incubated for another 2 h. The cells were fixed by 4% Paraformaldehyde for 30 min at room temperature, and incubated with peroxidase-coupled anti-BrdU-antibody (Sigma-Aldrich) for 90 min at 37°C. After being cleaned with PBS, the cells were incubated with DAPI (Beyotime, Shanghai, China) for 5 min at temperature. Subsequently, the BrdU-positive cells were counted under a fluorescence microscope (Olympus, Japan).

### Transwell Experiment

Cell migration and invasion were determined by the Transwell assay. Cells were put in the Transwell upper chamber at 2 × 10^4^ cells/well, and the lower chamber was supplemented with 600 μL culture medium containing 20% FBS and cultured at 37°C. Twelve hours later, the upper chamber cells were removed, immobilized with 4% paraformaldehyde, dyed with 0.1% crystal violet, dried, photographed and quantified. The invasion assay was the same as the migration assay, except that the upper chamber of the Transwell was pre-coated with Matrigel before adding the cells.

### Flow Cytometry

The Annexin V-FITC/PI Apoptosis Detection Kit (Yeason, Beijing, China) was used for evaluating the apoptosis of RCC cells. Briefly, the RCC cells were detached with 0.25% trypsin without EDTA and collected via centrifugation (300 g, at 4°C for 5 min). Next, the cells were washed twice with pre cooled PBS and centrifuged at 4°C for 5 min (300 g). 100 μL 1 × Binding buffer was used to suspend the cells, followed by incubating with 5 μL Annexin V-FITC and 10 μL PI stabilizing solution. After a 10-min incubation at room temperature, 400 μL 1 × Binding Buffer was added and the apoptosis was detected by flow cytometry at 488 nm (BD Biosciences, Franklin Lakes, NJ, United States).

### Real-Time Quantitative PCR

Total RNA was separated from 786-O and A-498 cells by applying the Trizol reagent. RNA was reversely transcribed into cDNA with the PrimeScript™ RT Reagent kit (Invitrogen, Shanghai, China) per the manufacturer’s guidelines. qPCR was implemented using the Bio-Rad CFX96 quantitative PCR system and SYBR, with pre-denaturation at 95°C for 5 min, followed by denaturation at 95°C for 15 s and annealing at 60°C for 30 s. U6 served as an endogenous control of miR-320a, and GAPDH was that of HECTD2 and HIF-1α. The 2 ^(−ΔΔCt)^ method was adopted for statistics. Each test was done three times. Primer sequences are exhibited in Table 3.

**TABLE 3:** 

Primer sequence of gene name (5’→3′)	
HECTD2	forward: GTA​GGG​GAA​GCT​GGT​TTG​GA reverse: CTT​GTA​ACA​GCA​GGG​AGG​GA
HIF-1α	forward: TCC​AAG​AAG​CCC​TAA​CGT​GT reverse: TGA​TCG​TCT​GGC​TGC​TGT​AA
miR-320a	forward: CAGTGCAGGGTCCGAGGT reverse: AAC​ACG​TGA​AAA​GCT​GGG​TTG​AG
U6	forward: TGC​GGG​TGC​TCG​CTT​CGG​CAG​C reverse: CCA​GTG​CAG​GGT​CCG​AGG​T
GAPDH	forward: AGA​GGC​AGG​GAT​GAT​GTT​CTG reverse: GAC​TCA​TGA​CCA​CAG​TCC​ATG​C

### Western Blot

The cells were treated, and the culture medium was removed. Then, the protein lysate (Roche) was added for total protein separation. 50 μg total protein was subjected to 12% polyacrylamide gel and went through 2 h of electrophoresis at 100 V. It was then transferred to polyvinylidene fluoride (PVDF) membranes. After being blocked with 5% skimmed milk at RT for 1 h, the membranes were rinsed with TBST three times (10 min each) and incubated with the primary antibodies (1:1,000) of anti-Bax (ab182734), anti-P53 (ab32389), anti-Caspase3 (ab32351), anti-P21 (ab109199), anti-HIF-1α (ab179483), anti-HECTD2 (ab249770), anti-E-cadherin (ab40772), anti-Vimentin (ab92547), and anti-N-cadherin (ab76011) overnight at 4°C. After washing the membranes with TBST, we incubated them with horseradish peroxidase (HRP)-labeled Goat anti-Rabbit IgG (ab205718) (1:2,500) for 1 h at RT. The above antibodies were obtained from Abcam (Cambridge, United Kingdom). Afterward, the membranes were cleaned with TBST 3 times (10 min each). Finally, the ECL chromogenic agent (Millipore, Bedford, MA, United States) was employed for exposure and imaging with a membrane scanner.

### Dual-Luciferase Reporter Assay

PmirGLO vectors (Promega, United States) containing miR-320a sequences with wild-type or mutant binding locations for HECTD2 (HECTD2-WT, HECTD2-MT) were adopted. 786-O and A-498 cells were co-transfected with miR-320a mimics and the luciferase vectors. Detection of luciferase activity was made with a Dual-luciferase Reporter Gene Assay System (Promega, United States).

### RNA Immunoprecipitation

RIP was implemented with Anti-AGO2 (#03-110, Millipore, Germany) by applying the Magna RIP RNA binding protein immunoprecipitation kit (Millipore, Germany). The RNA binding complex was analyzed by qRT-PCR. Anti-IgG served as an isotype control.

### Tumor Formation in Nude Mice

BALB/c-nu nude mice (six-week-old) were chosen to construct an *in vivo* tumorigenic model. Logarithmic growth phase 786-O and A-498 cells were obtained by 0.25% trypsin and resuspended in the serum-free medium to fabricate single-cell suspensions (3 × 10^7^ cells/mL). Next, 0.1 ml cell suspension was injected subcutaneously into the left forelimb armpit of each nude mouse (70 mice in total, 10 mice in each group). After injection, tumors were taken from mice under anesthesia on day 7, 14, 21, 28, and 35, respectively and weighed. The longest diameter of the tumor 1) and the shortest diameter perpendicular to it 2) were monitored with vernier calipers, and the tumor volume V (mm^3^) = 0.5 × a × b^2^. All experiments were authorized by the Animal Experimentation Ethics Review Committee of the Sichuan Provincial People’s Hospital. Experiments were implemented strictly following the National Institutes of Health Guidelines for the Care and Use of Laboratory Animals (NIH Publication No. 8023).

### TdT-Mediated dUTP Nick End Labeling Staining

As described previously, paraffin sections of tumor tissues were made, dewaxed with toluene, and then dehydrated with different gradients of ethanol following the standard procedure. TUNEL staining of tumor tissue was then performed using a fluorescent assay kit (Cat# 11684817910, Roche, Shanghai, China) according to the manufacturer’s guidelines.

### Immunohistochemistry

RCC tissue specimens were sectioned by conventional paraffin embedding (4 μM), dewaxed with conventional xylene, hydrated with gradient ethanol, and inactivated with 3% H_2_O_2_ for 10 min. Microwave repair was made by applying 0.01 mol/L sodium citrate buffer (pH = 6.0, 15 min). Afterward, the sections were closed with 5% bovine serum albumin (BSA) for 20 min, followed by incubation with the Ki67 antibody (ab15580, Abcam, MA, United States) overnight at 4°C. On day 2, the Goat-anti-Rabbit IgG was added and incubated at RT for 20 min, cleaned with PBS and imaged with DAB. The sections were inhibited by hematoxylin, dehydrated, and transparentized, and the closure was observed under the microscope.

### Tissue Immunofluorescence

Paraffin tissue sections were dewaxed and hydrated. After antigen repair, the sections were sealed and maintained with the primary antibodies, including anti-HIF-1α (ab179483, Abcam) and anti-HECTD2 (HPA037767, Sigma-Aldrich). After being washed with PBS, the Goat polyclonal Secondary Antibody to Rabbit IgG-H&L (Alexa Fluor® 488) (ab150077) was added for incubation. Afterward, 4,6 diamidinyl-2-phenyl indole (DAPI) nuclear staining was conducted, and the sections were blocked, observed, and photographed. Three sections were chosen from each specimen, and five non-overlap high-power fields of cancer tissues were randomly chosen from each section. The number of HIF-1α and HECTD2 staining positive cells in each field of view was counted using Image-Pro Plus image analysis software, and the number of HIF-1α and HECTD2 staining positive cells/mm^2^ was calculated.

### Statistical Analysis

SPSS22.0 statistical software (SPSS Inc., Chicago, IL, United States) was employed for statistical analysis. All data were presented as mean ± SD. Measurement data were expressed as mean ± standard deviation (x ± s), and enumeration data were expressed as four tables (or percentages). χ^2^ was adopted to compare differences between the two groups. One-way ANOVA was employed for the multi-factor comparison, and the comparison between two groups was made by *t* test. *p* < 0.05 represented statistical significance.

## Results

### Expression and Prognosis of HIF-1α and HECTD2 in RCC

**FIGURE 1:**
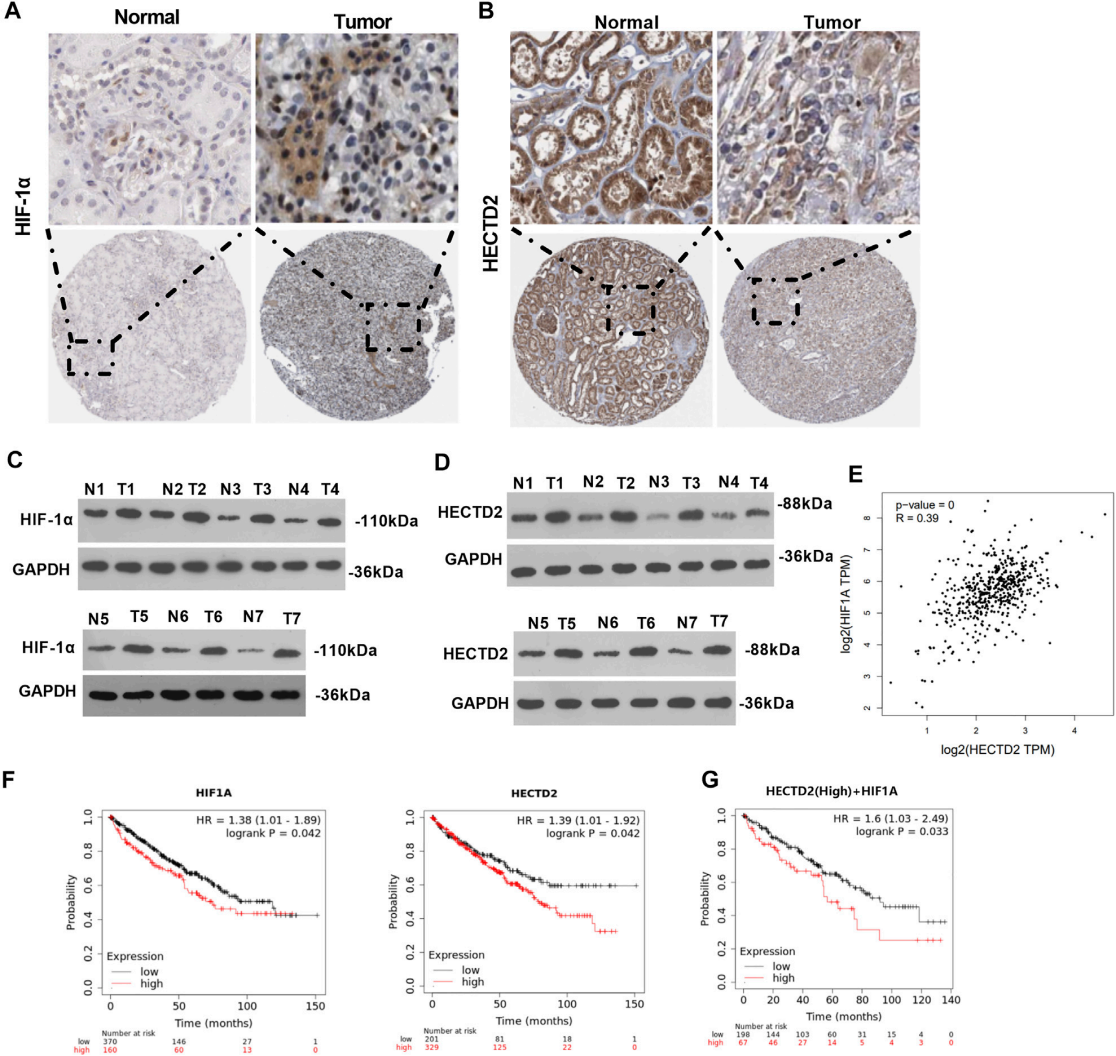
Expression and prognosis of HIF-1α and HECTD2 in RCC **(A,B)**. The number of HIF-1α and HECTD2 positive cells in RCC tissue and normal paracancerous tissues was analyzed in the Human Protein Atlas database (https://www.proteinatlas.org/). **(C,D)**: HIF-1α and HECTD2 protein profiles in 7 cases of RCC tissues (T) and normal paracancerous tissues (N) were compared by WB. **(E)** The online database GEPIA was used for analyzing the expression relationship in KIRC (http://gepia.cancer-pku.cn/). **(F,G)**: Prognostic analysis of HIF-1α and HECTD2 in RCC was performed on Kaplan-Meier Plotter (http://kmplot.com/analysis/).

To investigate whether HIF-1α and HECTD2 contribute to RCC progression, we consulted the database (https://www.proteinatlas.org/) and discovered that HIF-1α and HECTD2 levels were both highly expressed in RCC tissues (Figures 1A,B). Also, WB results uncovered that HIF-1α and HECTD2 expression levels were significantly higher in tumor tissues of RCC patients than those in corresponding surrounding non-tumor tissues (Figures 1C,D). More importantly, Fuhrman grade in patients with higher profiles of HIF-1α and HECTD2 was generally staged III + IV and tumor stage tended to be pT2/T3 (Tables 1, 2). Besides, there is a positive relationship between HIF-1α expression and HECTD2 expression in KIRC (analyzed by GEPIA) (Figure 1E). Meanwhile, patients with high expression of HIF-1α and HECTD2 had worse prognosis compared with those with low expression of HIF-1α and HECTD2. Additionally, those patients with higher HECTD2+higher HIF-1α had poorer overall survival of those with higher HECTD2+lower HIF-1α (*p* = 0.032) [data analyzed via Kaplan-Meier Plotter (http://kmplot.com/analysis/)] (Figures 1F,G). These results prompted that HIF-1α and HECTD2 were associated with the malignant phenotype of RCC cells and might exert carcinogenic effects.

### Impacts of Overexpressing HECTD2 on the Malignant Phenotypes of RCC *in vitro*

**FIGURE 2:**
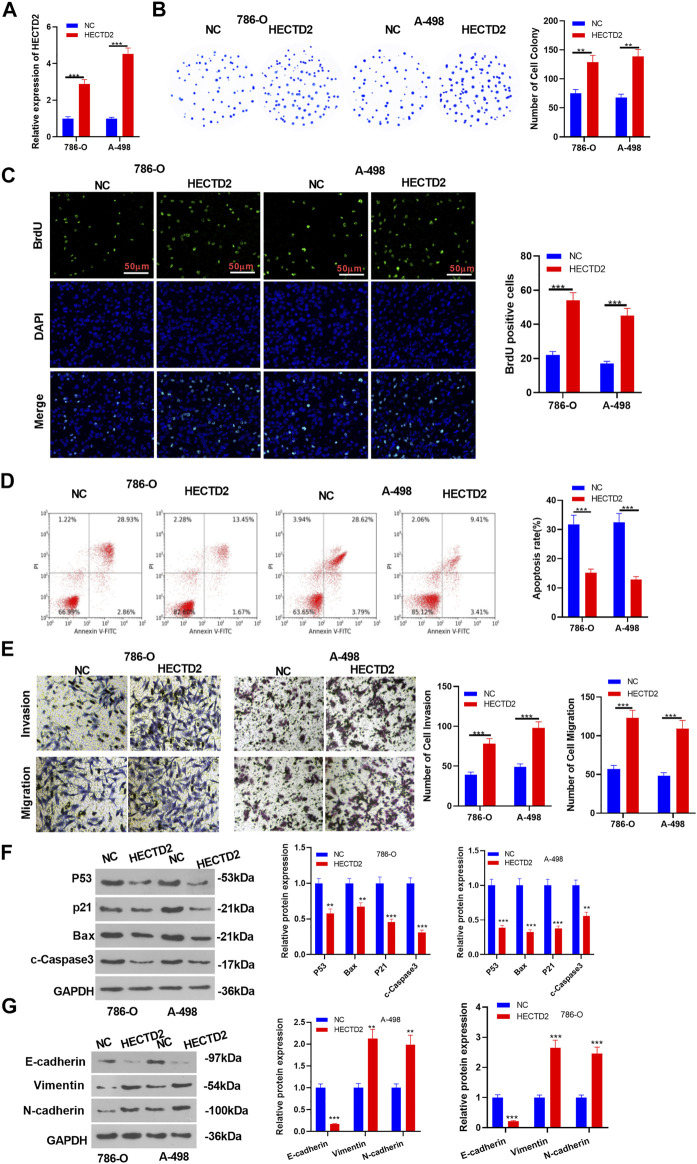
Effect of HECTD2 overexpression on the malignant phenotype of RCC. Overexpression models of HECTD2 were constructed in 786-O and A-498 cell lines. **(A)**: RT-qPCR was implemented to testify the HECTD2 expression. **(B–D)**: RCC cell proliferation and apoptosis were determined by the colony formation experiment, BrdU assay and flow cytometry, respectively. **(E)** RCC cell migration and invasion were assessed by Transwell assay. **(F,G)**: WB was conducted to examine the expression of P53, Bax, p21, c-Caspase3, E-cadherin, Vimentin, and N-cadherin. **p < 0.01, ***p < 0.001 vs. NC group. *N* = 3. Scale bar = 50 μm.

We constructed the HECTD2 overexpression model in 786-O and A-498 cell lines and tested the transfection effect by RT-qPCR to probe the influence of HECTD2 on the malignant progression of RCC (*p* < 0.05, Figure 2A). Meanwhile, the detection of RCC cell proliferation and apoptosis was made using the colony formation experiment, BrdU assay and flow cytometry, respectively. Interestingly, overexpressing HECTD2 significantly motivated proliferation and bridled apoptosis in RCC (*p* < 0.05, Figures 2B–D). Additionally, WB results illustrated that the pro-apoptotic proteins P53, Bax, p21, and c-Caspase3 were significantly down-regulated after HECTD2 overexpression (*p* < 0.05, Figure 2E). Besides, Transwell assay results testified that overexpressing HECTD2 significantly facilitated RCC cell migration and invasion (*p* < 0.05, Figure 2F). Also, WB results signified that overexpressing HECTD2 significantly repressed the expression of the epithelial marker E-cadherin and elevated the levels of interstitial markers Vimentin and N-cadherin (*p* < 0.05, Figure 2G). These findings confirmed that overexpressing HECTD2 intensified the malignant progression of RCC.

### Influence of HECTD2 Overexpression on the Malignant Phenotype of RCC *in vivo*

**FIGURE 3:**
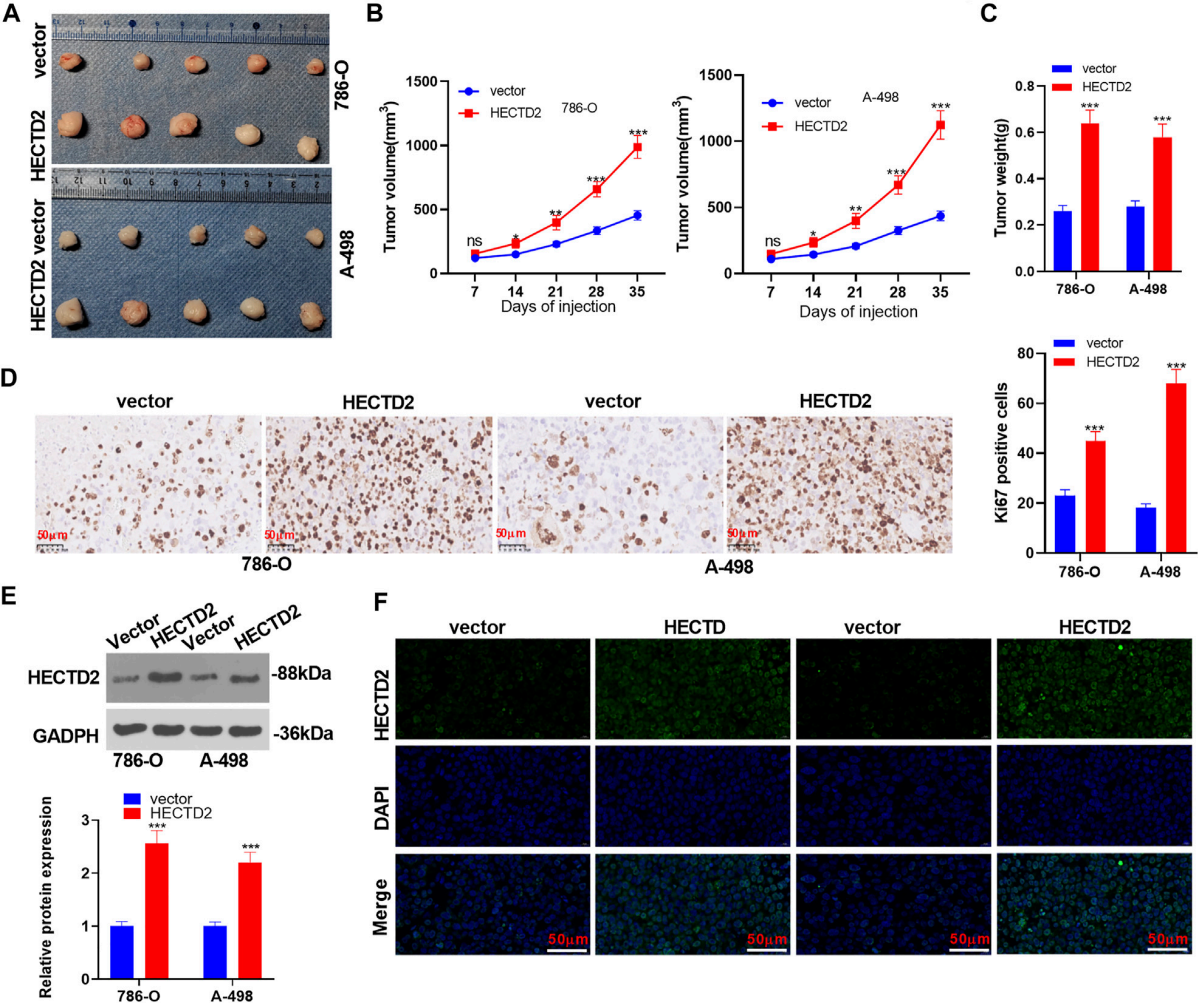
Influences of overexpressing HECTD2 on the malignant phenotypes of RCC *in vivo*. The HECTD2 overexpression model was established in 786-O and A-498 cells, which were then used for constructing xenografted tumor model in nude mice. The mice were sacrificed at the 35th day after cell transplantation. **(A–C)**: Tumor volume and weight. **(D)**: The number of Ki67-positive cells in 786-O and A-498 cells was evaluated by IHC. **(E)**: The profile of HECTD2 in the formed tumor tissues was tested by WB. **(F)**: The fluorescence intensity of HECTD2 was tested by tissue immunofluorescence. ns *p* > 0.05, *p < 0.05, **p < 0.01, ***p < 0.001. *N* = 5. Scale bar = 50 μm.

We constructed a HECTD2 overexpression model in 786-O and A-498 cells to testify the influence of HECTD2 on tumor growth *in vivo* and found that overexpressing HECTD2 significantly heightened tumor volume and weight (*p* < 0.05, Figures 3A–C). Also, IHC revealed a substantial elevation in the Ki67-positive cell number in 786-O and A-498 cells after HECTD2 overexpression (*p* < 0.05, Figure 3D). Moreover, WB results demonstrated that overexpressing HECTD2 in 786-O and A-498 cells significantly facilitated the HECTD2 profile in the transplanted tumor tissues (*p* < 0.05, Figure 3E). The tissue immunofluorescence data exhibited that the fluorescence intensity of the HECTD2 group was more obvious than that of the control group (*p* < 0.05, Figure 3F). These results added to evidence that overexpressing HECTD2 boosted RCC cell growth.

### HIF-1α Expedited the Expression of HECTD2 and Its Malignant Phenotype

**FIGURE 4:**
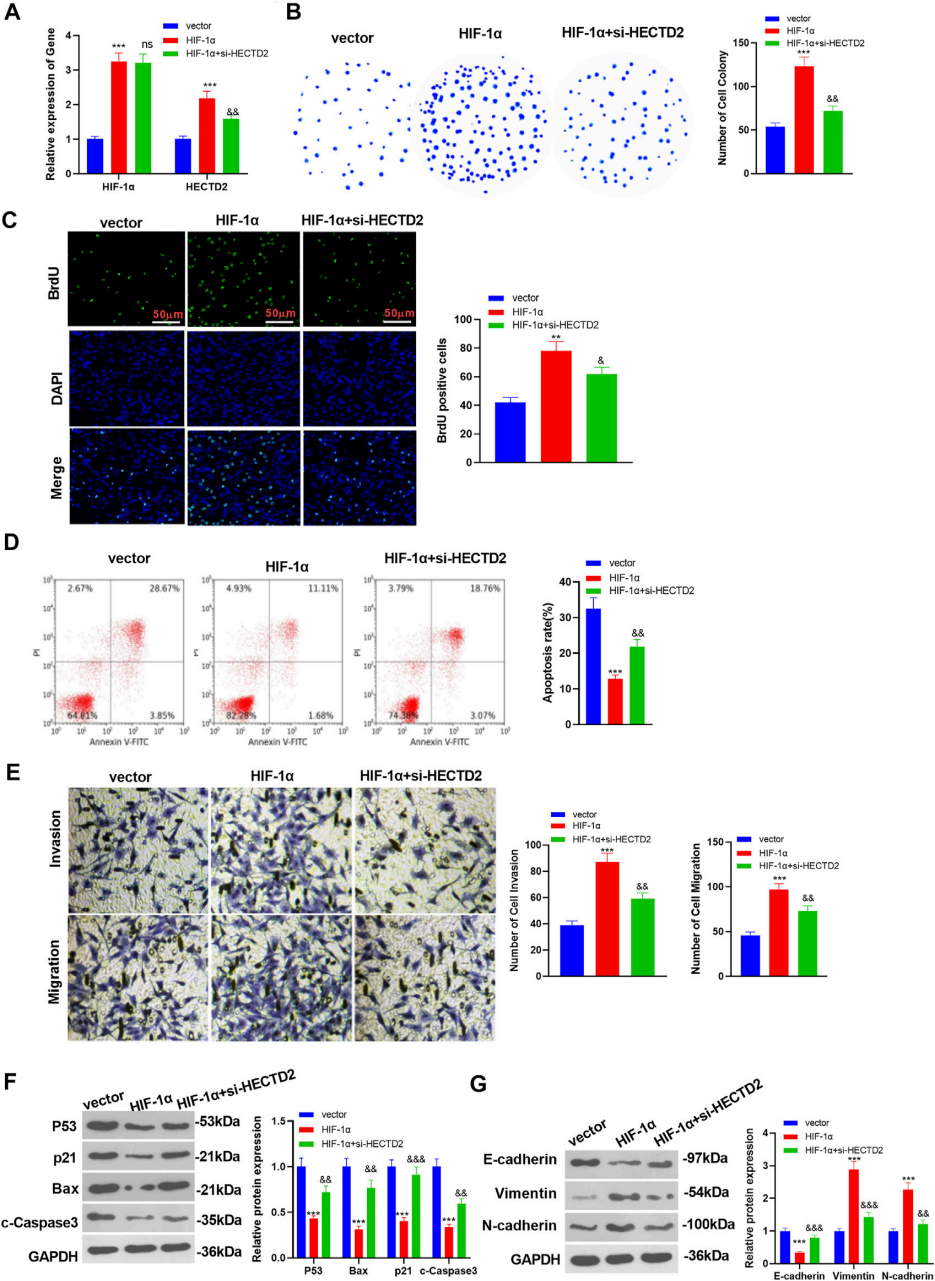
HIF-1α heightened the expression of HECTD2 and its malignant phenotype. HECTD2 was knocked down in 786-O cells overexpressing HIF-1α. **(A)**: RT-qPCR was carried out to monitor the levels of HIF-1α and HECTD2. **(B–D)**: RCC cell proliferation and apoptosis were checked using the colony formation experiment, BrdU assay and flow cytometry, respectively. **(E)**: Transwell assay was conducted to measure RCC cell migration and invasion. **(F,G)**: The expression of P53, Bax, p21, c-Caspase3, E-cadherin, Vimentin, and N-cadherin was compared by WB. ***p < 0.001 (vs. vector group). ns *p* > 0.05, && *p* < 0.01 (vs. HIF-1α group). *N* = 3. Scale bar = 50 μm.

To ascertain the function of HIF-1α and HECTD2 in RCC evolvement, we knocked down HECTD2 on the basis of HIF-1α overexpression in 786-O cells and monitored the expression of HIF-1α and HECTD2 using RT-qPCR. It turned out that HIF-1α and HECTD2 were up-regulated after HIF-1α overexpression. However, compared with the HIF-1α group, the HIF-1α expression in the HECTD2 group was not changed, while HECTD2 was evidently down-regulated (*p* < 0.05, Figure 4A). The colony formation, BrdU assay and flow cytometry were employed to examine RCC cell proliferation and apoptosis. The results unveiled that overexpressing HIF-1α facilitated the proliferation and impeded the apoptosis of RCC. Nevertheless, the findings of the HIF-1α + si-HECTD2 group were exactly opposite to those of the HIF-1α group (*p* < 0.05, Figures 4B–D). Meanwhile, Transwell assay results clarified that HIF-1α overexpression dramatically strengthened RCC cell invasion and migration. In contrast, RCC cell migration and invasion were significantly hampered in the HIF-1α + si-HECTD2 group (vs. the HIF-1α group) (*p* < 0.05, Figure 4E). WB exhibited that the expression of pro-apoptotic proteins P53, Bax, p21 and c-Caspase3 were suppressed after overexpressing HIF-1α. However, the profiles of these proteins were heightened in the HIF-1α + si-HECTD2 group (vs. the HIF-1α group) (*p* < 0.05, Figure 4F). Also, WB results confirmed that HIF-1α overexpression significantly hampered the expression of E-cadherin and elevated the levels of Vimentin and N-cadherin (vs. the control group). The addition of si-HECTD2 to HIF-1α subverted the effect of HIF-1α (*p* < 0.05, Figure 4G). These results manifested that up-regulating HIF-1α in 786-O cells resulted in the elevation of HECTD2 profiles and the aggravation of RCC.

### Effects of the HIF-1α/HECTD2 Axis on RCC Cell Growth *in vivo*

**FIGURE 5:**
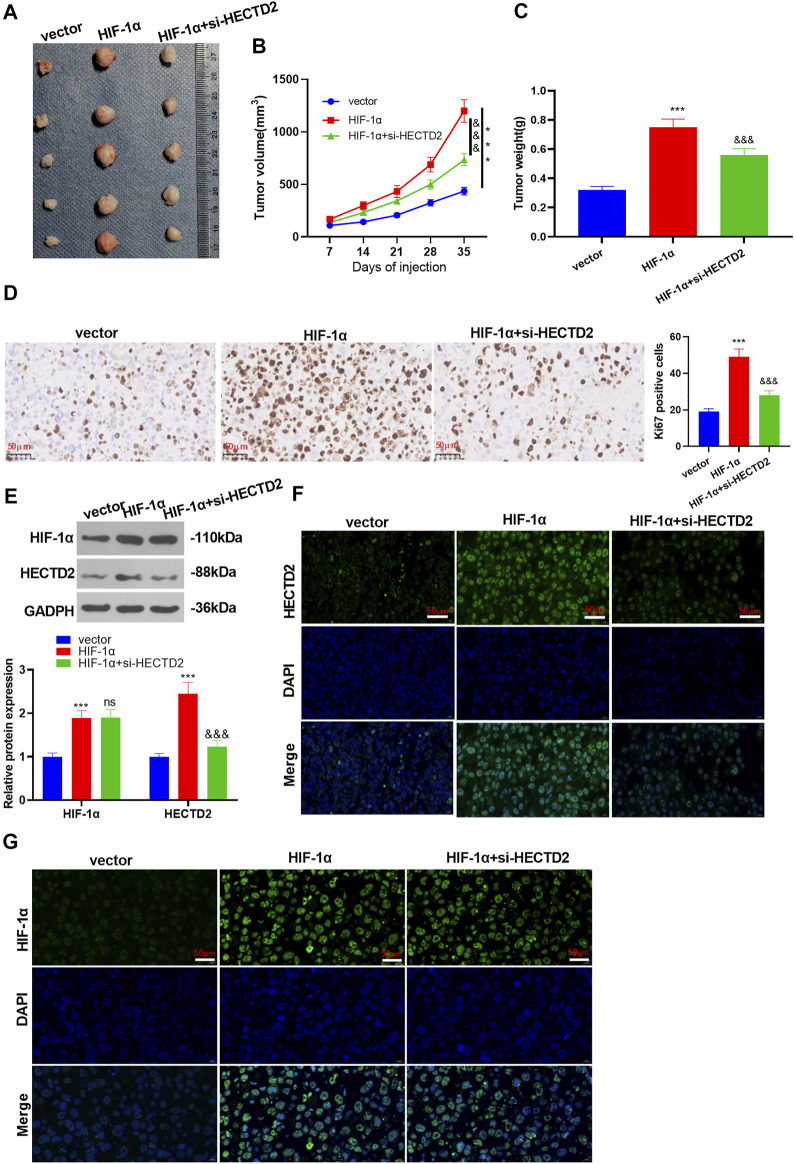
Impacts of the HIF-1α/HECTD2 axis on RCC cell growth *in vivo.* HIF-1α overexpression and/or HECTD2 knockdown cell model was used for constructing xenografted tumor model in nude mice. The mice were sacrificed at the 35th day after cell transplantation. **(A–C)**: Tumor volume and weight. **(D)**: IHC was adopted to count the number of Ki67-positive cells in 786-O and A-498. **(E)**: The profiles of HIF-1α and HECTD2 in 786-O and HECTD2 cells were monitored by WB. **(F,G)**: The fluorescence intensity of HIF-1α and HECTD2 was checked by tissue immunofluorescence. ns *p* > 0.05, *p < 0.05, **p < 0.01, ***p < 0.001. *N* = 5. Scale bar = 50 μm.

To further validate the impact of the HIF-1α/HECTD2 axis on tumor growth *in vivo*, we established HIF-1α overexpression and/or HECTD2 knockdown models in 786-O cells and discovered that overexpressing HIF-1α increased tumor volume and weight (vs. the vector group). However, HECTD2 knockdown suppressed RCC tumor volume and weight (vs. the HIF-1α group) (*p* < 0.05, Figures 5A–C). Also, immunohistochemical results exhibited evident facilitation in the Ki67-positive cell number in 786-O cells after HIF-1α overexpression, whereas the effect was reversed after HECTD2 knockdown (*p* < 0.05, Figure 5D). Besides, WB results illustrated that the profiles of HIF-1α and HECTD2 were elevated after HIF-1α overexpression in 786-O cells. However, compared with the HIF-1α group, the HIF-1α expression remained unchanged in the HIF-1α + si-HECTD2 group, but HECTD2 was significantly down-regulated (*p* < 0.05, Figure 5E). The results of tissue immunofluorescence exhibited that the fluorescence intensity of HIF-1α and HECTD2 was more pronounced in the HIF-1α group (vs. the vector group). Nevertheless, compared with the HIF-1α group, the fluorescence intensity of HIF-1α in the HIF-1α + si-HECTD2 group showed little change, but the fluorescence intensity of HECTD2 was dramatically weakened (*p* < 0.05, Figures 5F,G). These findings further confirmed the influence of HIF-1α overexpression on HECTD2 and RCC cell growth *in vivo*.

### miR-320a Targeted HECTD2

**FIGURE 6:**
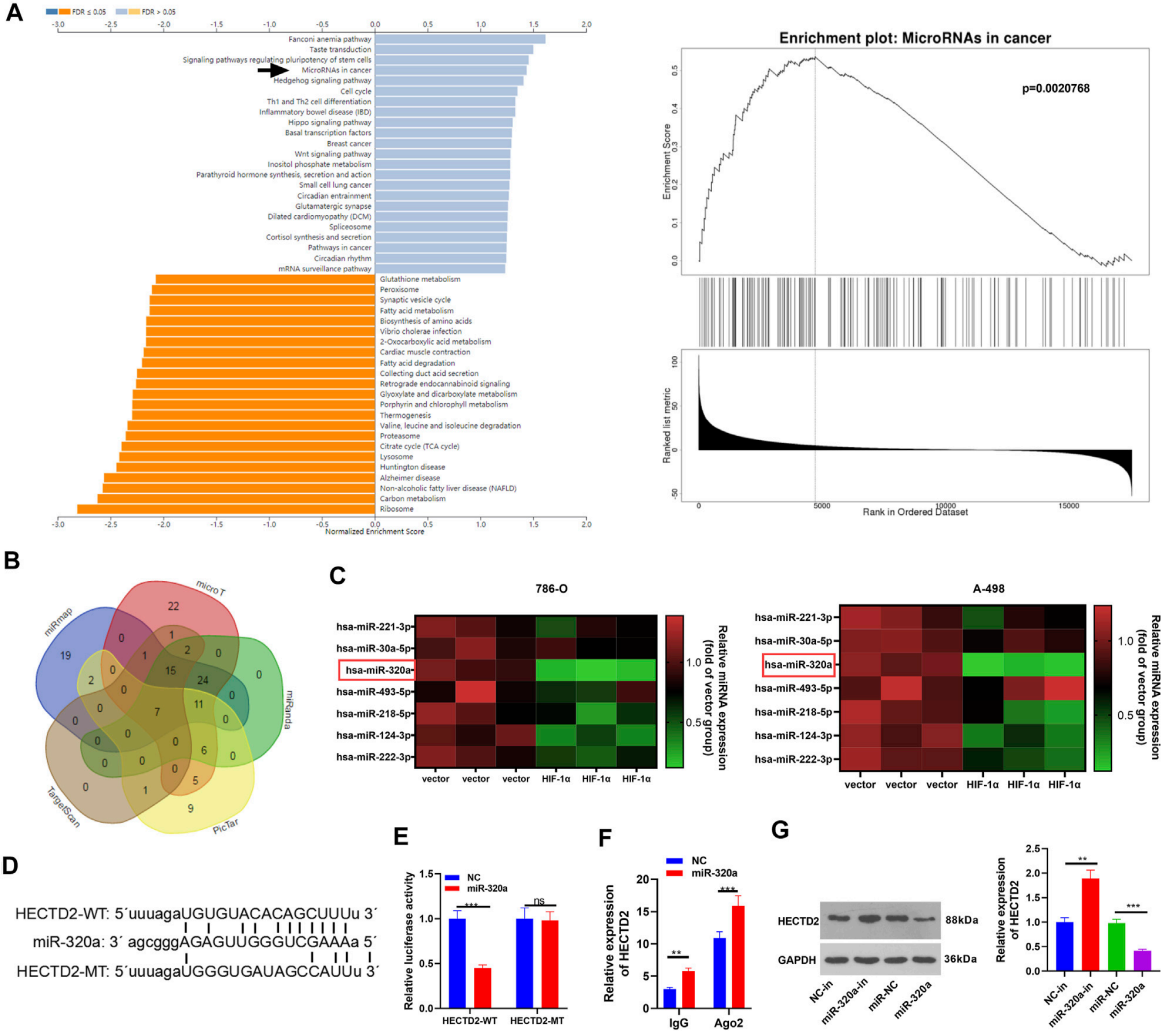
miR-320a targeted HECTD2. **(A)** The positive or negative related genes of HECTD2 in TGCA database was analyzed *via* LinkedOmics, and those genes were subjected to GSEA enrichment analysis. HECTD2 is potentially involved in a KEGG pathway of MicroRNAs in Cancer. **(B)**: Targetscan, miRmap, microT, PicTar and miRanda databases were utilized to search the miRNA target of HECTD2. Venn diagram was utilized to analyze the common target miRNAs of HECTD2. Seven common miRNAs were identified, including has-miR-221-3p, has-miR-30a-5p, has-miR-320a, has-miR-493-5p, has-miR-218-5p, has-miR-124-3p and has-miR-222-3p. **(C)**: RT-PCR was used for determining the seven miRNAs in 786-O and A-498 cells transfected with HIF-1α. **(D)**: HECTD2 contained the binding sites with miR-320a. **(E,F)**. Dual-luciferase reporter assay and RIP assay were applied to probe the binding of miR-320a to HECTD2. G: HECTD2 level in 786-O cells transfected with miR-320a mimics or miR-320a inhibitors was tested by WB. NS p > 0.05, **p < 0.01, ***p < 0.001. *N* = 3.

**FIGURE 7:**
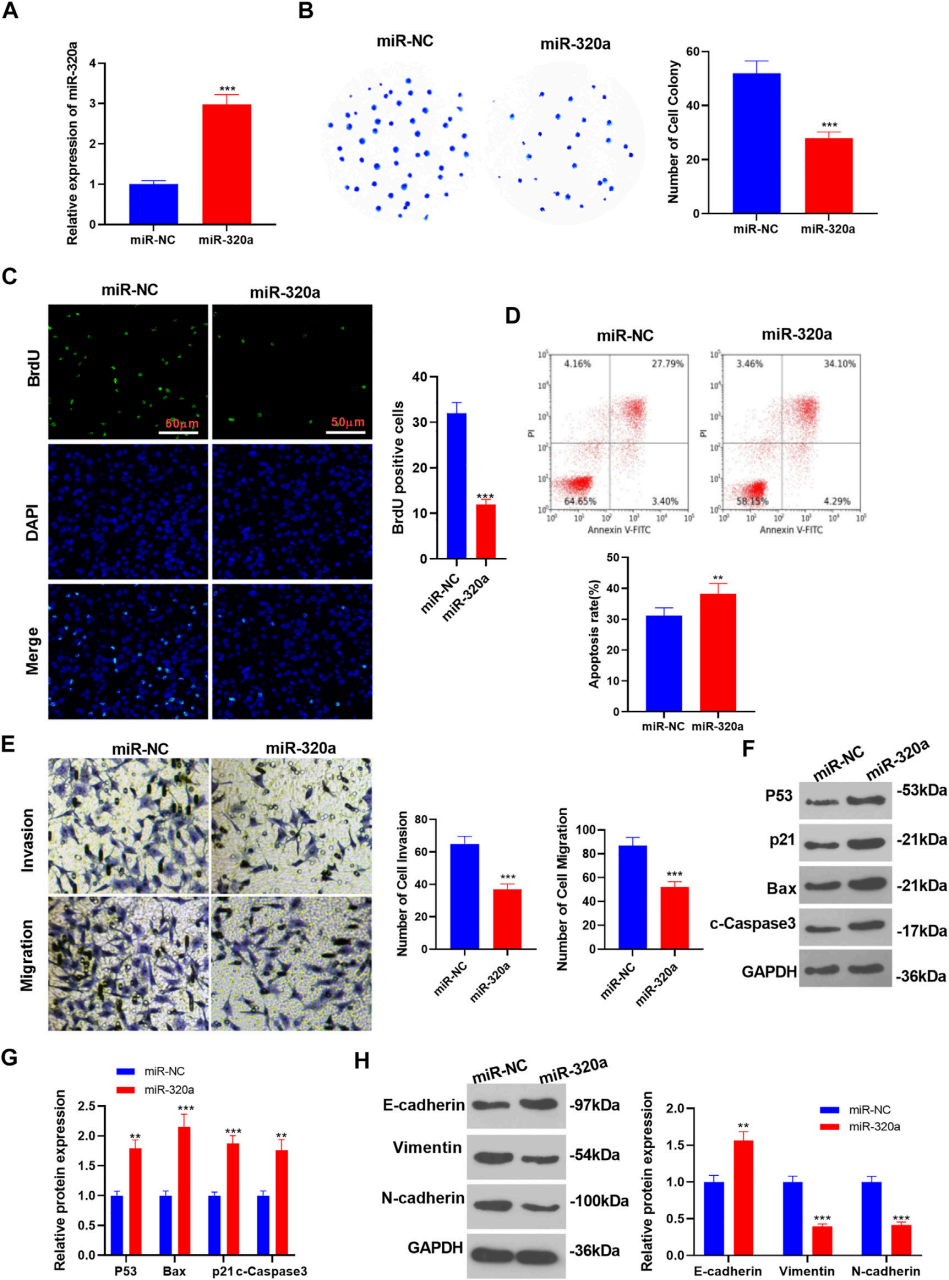
Influences of miR-320a on the malignant phenotype in RCC. miR-320a mimics were transfected into A-498 cells. **(A)**: The miR-320 profile was determined by RT-qPCR. **(B–D)**: RCC cell proliferation and apoptosis were checked by the colony formation assay, BrdU assay and flow cytometry, respectively. **(E)**: Transwell assay was implemented to verify RCC cell migration and invasion. **(F–H)**: WB was conducted to evaluate the expression of P53, Bax, p21, c-Caspase3, E-cadherin, Vimentin, and N-cadherin. **p < 0.01, ***p < 0.001. *N* = 3.

For analyzing the upstream mediator of HECTD2, we analyzed the positive or negative related genes of HECTD2 via LinkedOmics, and those genes were subjected to GSEA enrichment analysis. The results indicated that HECTD2 is involved in a KEGG pathway of MicroRNAs in Cancer (Figure 6A). Inspired by the miRNA-mRNA regulatory network, we adopted Targetscan, miRmap, microT, PicTar and miRanda to search the miRNA target of HECTD2 and discovered that HETCD2 had seven miRNAs as its common targets (Figure 7A). After that, we examined the profiles of these seven miRNAs in HIF-1α overexpressing cells by RT-PCR. It turned out that miR-320a was most significantly down-regulated (Figure 6C). The base binding sites between miR-320a and HECTD2 was shown in Figure 6D. The dual-luciferase reporter assay results demonstrated that miR-320a weakened the luciferase activity of HECTD2-WT, but it had no substantial effect on HECTD2-MUT (*p* < 0.05, Figure 6E). The RIP results demonstrated that miR-320a mimic transfection resulted in a higher amount of HECTD2 deposited in the Ago2 antibody group than that in the IgG group, implying that HECTD2 was bound to Ago2 via miR-320a (*p* < 0.05, Figure 6F). Moreover, the effect of modulating miR-320a on HECTD2 expression was examined by RT-qPCR. Interestingly, overexpressing miR-320a hampered the HECTD2 profile, while miR-320a knockdown showed the reverse (*p* < 0.05, Figure 6G). These findings testified that there was a targeting correlation between miR-320a and HECTD2.

### Impacts of miR-320a on the Malignant Phenotypes of RCC

To figure out the influences of miR-320a on HECTD2 and RCC evolvement, we transfected miR-320a mimics in A-498 cells and verified the transfection validity by RT-qPCR (*p* < 0.05, Figure 7A). Meanwhile, the results of the colony formation assay, BrdU assay and flow cytometry illustrated that overexpressing miR-320a restrained RCC cell proliferation and intensified apoptosis (*p* < 0.05, Figures 7B–D). Additionally, Transwell assay results manifested that RCC cell migration and invasion were curbed by miR-320a overexpression (*p* < 0.05, Figure 7E). Besides, WB results uncovered that the transfection of miR-320a mimics up-regulated P53, Bax, p21, and c-Caspase3 (vs. miR-NC group) (*p* < 0.05, Figures 7F,G). Moreover, WB results also signified that overexpressing miR-320a significantly facilitated E-cadherin expression and hampered the levels of Vimentin and N-cadherin (vs. the miR-NC group) (*p* < 0.05, Figure 7H). These results testified that transfection of miR-320a mimics in A-498 cells alleviated RCC progression.

### miR-320a Influenced RCC Growth and Epithelial-Mesenchymal Transition *via* HECTD2

**FIGURE 8:**
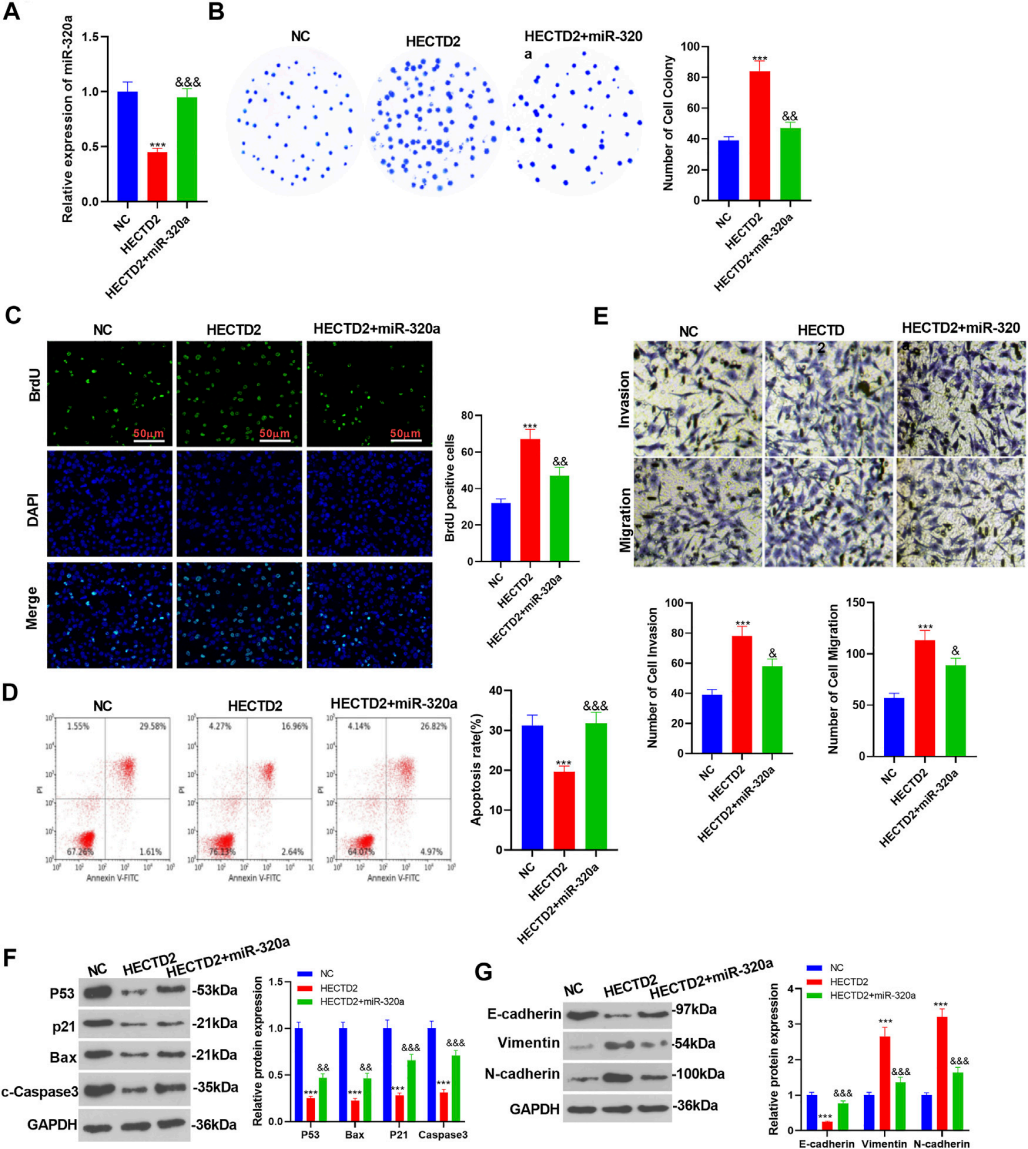
miR-320a affected RCC growth and EMT *via* HECTD2. Transfection of miR-320a mimics was made in 786-O cells with overexpressed HECTD2. **(A)**. RT-qPCR was implemented to examine miR-320 expression. **(B–D)**: The colony formation assay, BrdU assay, and flow cytometry were applied to assess RCC cell proliferation and apoptosis. RCC cell migration and invasion were tested by Transwell assay. **(F,G)**: The profiles of P53, Bax, p21, c-Caspase3, E-cadherin, Vimentin, and N-cadherin were compared by Transwell assay. ***p < 0.001, & *p* < 0.05, && *p* < 0.01, &&& *p* < 0.001. *N* = 3.

To explore the specific mechanism by which miR-320a affected RCC growth and EMT, we transfected miR-320a mimics in 786-O cells overexpressing HECTD2 and assayed the transfection validity by applying RT-qPCR. As a result, compared with the NC group, miR-320a was down-regulated in the HECTD2 group and was up-regulated in the miR-320a group (*p* < 0.05, Figure 8A). The colony formation assay, BrdU assay and flow cytometry results exhibited enhanced RCC cell proliferation and significantly repressed apoptosis in the HECTD2 group compared to the NC group, while the transfection of miR-320a mimics exerted the opposite function (*p* < 0.05, Figures 8B–D). Meanwhile, Transwell assay results showed that RCC migration and invasion were significantly enhanced in the HECTD2 group compared with the NC group, while they were attenuated after the miR-320a mimic transfection (*p* < 0.05, Figure 8E). Also, WB exhibited that the protein profiles of P53, Bax, p21 and c-Caspase3 were evidently declined in the HECTD2 group (vs. the NC group). Nevertheless, these proteins were significantly up-regulated in the HECTD2 +miR-320a group (vs. the HECTD2 group) (*p* < 0.05, Figure 8F). Moreover, WB results illustrated that the E-cadherin profile was impeded, while Vimentin and N-cadherin were significantly up-regulated in the HECTD2 group (vs. the NC group). The HECTD2+miR-320a group had completely opposite results to the HECTD2 group (*p* < 0.05, Figure 8G). These findings manifested that HECTD2 enhanced RCC cell growth and EMT in 786-O cells, but transfection with miR-320a mimics significantly alleviated RCC (Graphical Abstract).

## Discussion

RCC is among the most familiar tumors of the genitourinary system. Therefore, it’s urgent to research the carcinogenesis and progressive mechanism of RCC and to search for potential molecular biomarkers and therapeutic targets (Chow et al., 2010). Here, we discovered that HIF-1α and HECTD2 were enhanced in RCC, and *in-vivo* studies testified that overexpressing HECTD2 and HIF-1α hampered apoptosis and induced tumor growth of RCC cells. Further studies illustrated that HIF-1α contributed to RCC progression by inhibition of miR-320a, an upstream regulator of HECTD2.

A major character of cancer environment, hypoxia is often caused by abnormal tumor growth when tumor cells are lack of oxygen supply (Jahanban-Esfahlan et al., 2018). HIF-1α is not only a key nuclear transcription factor in the tumor hypoxia, but also an upstream transcriptional regulator of genes associated with tumor cell proliferation, apoptosis, neovascularization, invasion and metastasis (Lin et al., 2017; Knight and Stanley, 2019). Emerging researches have reported that HIF-1α overexpression contributes to poor prognosis and is a therapy target in diversified cancers (Yang et al., 2014; Egawa et al., 2021; Yang et al., 2021). Many strategies have been used in restraining tumor progression and chemoresistance by targeting HIF-1α (Luo et al., 2021). In RCC, HIF-1α upregulation enhances proliferation, migration and survival by mediating metabolic reprogramming (de Carvalho et al., 2021). Transglutaminase 2 (TGase 2) promoted angiogenesis by inducing p53 degradation, thus activating HIF-1α/VEGF2R pathway (Lee et al., 2020). In addition, HIF-1α has a role in regulating RCC cell glycolysis. HIF-2α, another Hypoxia-inducible factor, affects antigen presentation, interferon signalling and CD8 T cell infiltration and activation (Hoefflin et al., 2020). Presently, we discovered that HIF-1α was up-regulated in RCC tissues and induces enhanced RCC cell proliferation, growth, migration and reduced apoptosis.

As a vital miRNA, miR-320a has been proved to have significant values in colorectal cancer (Zhang et al., 2021a), epithelial ovarian cancer (Zhang et al., 2021b), nasopharyngeal cancer (Xing et al., 2021), melanoma (Fu et al., 2020). More importantly, it has been reported that various miRNAs, such as miR-155 (Meng et al., 2021), miR-224-5p (Qin et al., 2021), miR-30b-5p (Zhang et al., 2021c), etc., are involved in the development of RCC by targeting different genes. Interestingly, miR-320a has been found downregulated in RCC tissues and cell lines (data from TCGA), and it represses the proliferation, invasion via targeting FoxM1 (Zhao et al., 2018). This article revealed that overexpressing miR-320a hampered RCC proliferation, migration, invasion, and EMT and facilitated apoptosis, confirming that miR-320a was involved in RCC evolvement as a tumor suppressor.

miRNAs are dysregulated in cancers under hypoxia microenvironment, which are particularly associated with poor prognosis in cancer patients (Yang et al., 2020). Those miRNAs could be regulated at transcriptional and post-translational levels (Bandara et al., 2017). Recently, studies have revealed that hypoxia-stimulated long non coding RNAs (lncRNAs) inhibit miRNAs in cytoplasm and represses the expression of the later (Li et al., 2021; Wan et al., 2021). Moreover, hypoxia promotes the released of extracellular vesicles (EVs), which are enriched with miRNAs, thus inducing apoptosis decrease and proliferation enhancement (Yin et al., 2021; Endzeliņš et al., 2018). In RCC, hypoxia induced upregulation of miR-346 and N-myc downstream-regulated gene 2 (NDRG2) inhibition in renal cancer cells. miR-346 represses NDRG2 and promotes the malignant behaviors of RCC cells (Su et al., 2020). As hypoxia-related transcription factors, HIF-1 and HIF-2 have been confirmed to play a dominant role in transcriptional gene regulation in hypoxia, including miRNAs (Serocki et al., 2018), and this mechanism is also involved in RCC progression (White and Yousef, 2010; Rustum et al., 2018). In this study, we found that HIF-1α overexpression significantly inhibits miR-320a expression, which suggests that HIF-1α potentially induces increased malignant phenotypes of RCC cells by suppressing miR-320a.

HECTD family members have been found associated with hypoxia microenvironment alteration. For instance, Hectd1 is regulated by insulin, heat, and hypoxia during embryogenesis in mice (D’Alonzo et al., 2019). Hypothermic oxygenated perfusion (HOP) prevents ischemia-reperfusion-mediated liver damage. The underlying mechanism revealed that both the DOC and HECT domains of HECTD3 induce polyubiquitination of TRAF3 at Lys138, and ubiquitinated TRAF3 promotes oxidative stress and inflammation (Zhou et al., 2021). Here, we showed that HECTD2 upregulation enhances RCC progression both *in vitro* and *in vivo*, and HIF-1α had a positives relationship with HECTD2 and increased its expression. To further clarify the mechanism of HECTD2, we searched its upstream target miRNAs and found a binding site shared by miR-320a and HECTD2. Furthermore, the dual-luciferase and RIP experiments confirmed the targeted binding association between the two. As a matter of fact, HECTD2 has been reported to exert a prominent role in tumor and is modulated by miRNA (Sun et al., 2014). What’s more, transfection of miR-320a mimics dampens RCC cells proliferation, migration, invasion followed by HECTD2 upregulation. These results showed that miR-320a affected RCC progression via directly targeting HECTD2.

Overall, our study confirmed that 1) HECTD2 is up-regulated in RCC and aggravates RCC progression; 2) miR-320a functions as a tumor suppressor in RCC by targeting HECTD2; 3) HIF-1α induces HECTD2 upregulation by repressing miR-320a. In summary, this study manifests a novel regulatory axis of HIF-1α/miR-320a/HECTD2 in RCC progression, which may be a viable therapeutic target for RCC. However, further experiments are needed for investigating the potential HIF-1α/miR-320a/HECTD2 axis in treating RCC *in vivo* and the downstream mechanism of HECTD2 in RCC.

## Data Availability

The original contributions presented in the study are included in the article/Supplementary Material, further inquiries can be directed to the corresponding authors.
